# Dental general anaesthetic trends among Australian children

**DOI:** 10.1186/1472-6831-6-16

**Published:** 2006-12-21

**Authors:** Lisa M Jamieson, Kaye F Roberts-Thomson

**Affiliations:** 1 Australian Research Centre for Population Oral Health, The University of Adelaide, South Australia 5005, Australia

## Abstract

**Background:**

Children receive dental general anaesthetic (DGA) care when standard dental treatment is not possible. Receipt of DGA care is resource-intensive and not without risk. This study examines trends in receipt of DGA care among Australian children.

**Methods:**

Child DGA data were obtained from the Australian Institute of Health and Welfare Hospital Morbidity Database for 1993–2004. Poisson regression modelling was used to examine DGA rates in relation to age, sex, Indigenous status, location, year and procedure.

**Results:**

There was a 3-fold increase in DGA rates from 1993–1994 (215.8 ± 2.9 per 100,000) to 2003–2004 (731.4 ± 5.3 per 100,000) (P < 0.001). Across all years, children who were aged 0–4 years, male or rural/remote-dwelling had higher DGA rates than their 5–9-year-old, female or metropolitan-dwelling counterparts respectively. There was a 7.0-fold increase in the rate of Indigenous admissions from 1993–1994 (116.5 ± 10.2 per 100,000) to 2003–2004 (806.6 ± 25.7 per 100,000). Extraction rates increased 4.9-fold from 1993–1994 (109.2 ± 2.9 per 100,000) to 2003–2004 (540.0 ± 4.5 per 100,000), while restoration rates increased 3.3-fold in the same observation period (139.5 ± 2.3 per 100,000 in 1993–1994 to 462.6 ± 4.2 per 100,000 in 2003–2004). For admissions in which one or more extractions were received, Indigenous rates were 47% greater than non-Indigenous rates after adjusting for other covariates.

**Conclusion:**

Child DGA rates in Australia are increasing. Children who are pre-school-aged, male, Indigenous or living in a rural/remote location are disproportionally represented among those receiving such care. There are higher rates of extractions as opposed to more conservative procedures, particularly among Indigenous children.

## Background

The proportion of children requiring dental care in many developed countries is decreasing [1]. Most children who do require such care are able to receive simple treatment in a dental chair, with local anaesthesia or sedation procedures being used if necessary. However, a small minority of children require dental care under general anaesthesia settings in a hospital situation. The most common reasons for dental general anaesthetic (DGA) care include the dental disease scenerio being too severe to warrant dental treatment under more conventional settings, behavioural problems of the child or medical/physical complications [2,3]. The advantage of oral rehabilitation under general anaesthesia is that it allows treatment in a single visit, provides immediate relief of pain and requires little to no co-operation by the child.

Children requiring DGA care are often of low socio-economic status or ethnic background [4], have received dental care under a general anaesthetic before [5] or have carers with poor oral health themselves [6]. There is some risk associated with DGA care, with its profile recently raised in the United Kingdom following a death that occurred whilst a patient was undergoing DGA care provided in a non-hospital setting [7].

DGA services are resource-intensive, both to the tax-payer and to carers of children undergoing such treatment. Hospitals providing DGA services are usually in large urban centers, meaning families living in rural/remote locations may face financial pressure incurred by time off from work, childcare, travel and accommodation. The strict procedures required before and after admission for DGA care, such as fasting before the procedure and the necessity for the child to stay overnight on occasion may place additional stress on the family unit, particularly if unfamiliar with hospital settings and protocols.

In Australia, a country with a 0–9-year-old population of over 2.6 million in 2002 [8], the demand for child DGA care is increasing. Waiting lists of up to two years exist in some jurisdictions [2]. Defining the scope and extent of the problem is a matter of some importance, both to inform the development of appropriate DGA service provision for children requiring such care and to reorient primary dental services so that the demand for treatment under a general anaesthetic might be reduced. This has public health implications for other countries also, particularly developing countries that are experiencing increasing urbanisation and rising levels of child caries prevalence.

We retrospectively examined hospital separation data collected at a national level in Australia from 1993–1994 to 2003–2004 with the purpose of exploring trends in receipt of child DGA care in relation to age, sex, Indigenous status, location and treatment type. The study population included children aged 0–9 years who had been admitted to hospital for receipt of dental care under a general anaesthetic. We aimed to test three hypotheses in this simple descriptive study: (i) DGA rates among children would be increasing; (ii) DGA rates would be higher among younger children, those identifying as Indigenous and those living in a rural/remote location and; (iii) there would be higher rates of extractions as opposed to more conservative procedures under DGA.

## Methods

Data on dental procedures received by children admitted to public and private hospitals across all Australian states and territories were accessed from the Australian Institute of Health and Welfare National Hospital Morbidity Database from 1 July 1993 until 30 June 2004. Data were collected for administrative purposes by hospital-employed dentists and recorded in standardised ICD-9+10-AM (International Statistical Classification of Diseases and Related Health Problems, 9th and 10th Revision, Australian Modification) codes, which are patient record codes used throughout Australian hospitals. Because all data were de-identified and collected primarily for administrative purposes, the Human Research Ethics Committee of the University of Adelaide did not consider ethical approval to be necessary for the secondary analysis of such data. The ICD-9+10-AM dental procedure codes pertaining to extractions or restorations were included. Demographic information was collected and included patients' age, sex, Indigenous status and residential location. Two age groups were considered: 0–4 years and 5–9 years. Indigenous status was defined by a child being Aboriginal, Torres Strait Islander or both, and was indicated by a carer upon admission. Separations with Indigenous status 'not stated' were excluded from the analyses.

Residential location was measured using the Rural, Remote and Metropolitan Areas (RRMA) classification, which is an index based on Statistical Local Areas (SLA) that allocates each SLA in Australia to a category based primarily on population numbers and an index of remoteness. 'Metropolitan' is defined as any capital city or other metropolitan area with a population of >100,000, 'rural' zones are those with a population ranging from 10–99,000 and 'remote' areas those with a population of <10,000. For the purposes of this study, 'rural' and 'remote' zones were combined.

Estimated resident population (ERP) counts of all demographic stratifications (sex, age, Indigenous status, location) for the years 1993–1994 to 2003–2004 were provided by the Australian Bureau of Statistics. Rates were generated by dividing the number of DGA admissions for a specified strata/procedure by the ERP of the same specified strata and multiplying by 100,000. We defined Poisson regression models to examine the strength of any independent association between child DGA rates and the covariates described above. Results from the Poisson regression models are presented as rate ratios with 95% confidence intervals (CI) to estimate the independent effect of each covariate on the child DGA rate. Data were analysed using Intercooled Stata 8.0.

## Results

**Figure 1:**
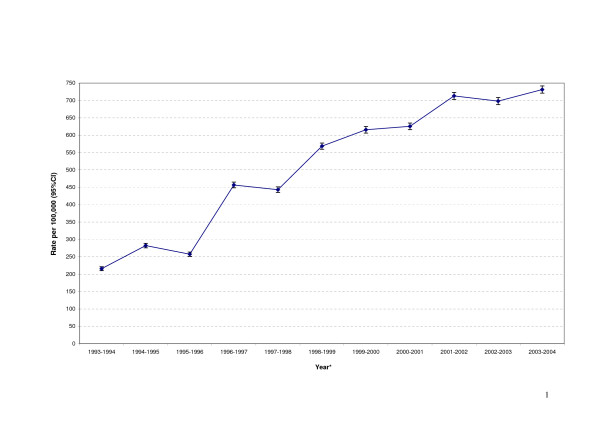
**DGA rates for 0–9-year-olds in Australia, 1993–2004**. *(P < 0.001): Poisson regression.

There was a 3-fold increase in child DGA rates from 1993–1994 (215.8 ± 5.7 per 100,000) to 2003–2004 (731.4 ± 10.3 per 100,000) (Figure 1). The steepest increase was noted between 1995–1996 and 1996–1997. The only significant decrease was observed between 1994–1995 and 1995–1996.

Across all years, children aged 0–4 years had higher DGA rates than their counterparts aged 5–9 years (Table 1). The greatest difference was noted in 1997–1998, when the rates of 0–4-year-olds was 1.3 times the rate of those aged 5–9 years. Across all years, males had higher DGA rates than females. The greatest difference was observed in 2003–2004, when male DGA rates were 774.3 ± 7.6 per 100,000 and female DGA rates were 686.2 ± 7.3 per 100,000. Indigenous child DGA rates were greater than non-Indigenous rates from 1997. There was a 7.0-fold increase in the rate of Indigenous admissions from 1993–1994 (116.5 ± 10.2 per 100,000) to 2003–2004 (806.6 ± 25.7 per 100,000). The greatest difference was observed in 1998–1999, when Indigenous DGA rates were 1.3 times those of their non-Indigenous counterparts. Rural/remote-dwelling children had higher DGA rates than their metropolitan-dwelling counterparts across all observed years. The admission rate for rural/remote-dwelling children was 776.4 ± 6.8 per 100,000 in 2003–2004 compared with 275 ± 3.4 per 100,000 in 1993–1994; a 2.8-fold increase. The DGA rates for procedures involving one or more restorations were greater than the rates for procedures involving one or more extractions until 1997–1998, after which the opposite was observed. Extraction rates increased 4.9-fold from 1993–1994 (109.2 ± 2.9 per 100,000) to 2003–2004 (540.0 ± 4.5 per 100,000), while restoration rates increased 3.3-fold in the same observation period (139.5 ± 2.3 per 100,000 in 1993–1994 to 462.6 ± 4.2 per 100,000 in 2003–2004).

**Table 1:** Child DGA rates per 100,000 by demographic and dental procedure characteristics, 1993–1994 to 2003–2004 (SE in brackets)

	1993–1994	1994–1995	1995–1996	1996–1997	1997–1998	1998–1999	1999–2000	2000–2001	2001–2002	2002–2003	2003–2004
Total	215.8 (2.9)	282.2 (3.3)	257.4 (3.1)	456.9 (4.2)	443.2 (4.1)	568.6 (4.6)	615.6 (4.8)	625.6 (4.9)	713.1 (5.2)	698.3 (5.2)	731.4 (5.3)
											
Age-group											
0–4 years	242.1 (4.3)*	312.2 (4.9)*	276.0 (4.6)*	505.8 (6.2)*	501.6 (6.2)*	618.4 (6.9)*	651.1 (7.1)*	652.0 (7.1)*	726.0 (7.4)*	721.1 (7.3)*	762.1 (7.4)*
5–9 years	189.2 (3.8)	251.8 (4.4)	238.6 (4.3)	408.4 (5.6)	385.9 (5.4)	520.4 (6.2)	581.7 (6.6)	600.5 (6.6)	690.1 (7.3)	674.2 (7.3)	698.9 (7.5)
											
Sex											
Male	227.4 (4.1)*	299.0 (4.7)*	266.3 (4.5)	478.9 (6.0)*	465.8 (5.9)*	600.8 (6.7)*	641.2 (6.9)*	657.9 (7.0)*	747.7 (7.4)*	731.2 (7.4)*	774.3 (7.6)*
Female	203.5 (4.0)	264.5 (4.6)	248.0 (4.4)	433.8 (5.8)	419.5 (5.7)	543.7 (6.5)	588.6 (6.8)	591.5 (6.8)	676.6 (7.2)	663.6 (7.2)	686.2 (7.3)
											
Location											
Metropolitan	155.8 (2.1)*	227.2 (2.6)*	217.4 (3.0)*	416.9 (4.0)*	383.2 (4.2)*	538.6 (4.5)*	565.6 (4.6)*	585.6 (4.6)*	683.1 (5.0)*	668.3 (6.7)*	686.4 (6.8)*
Rural/Remote	275.8 (3.4)	337.2 (3.7)	297.4 (4.5)	496.9 (5.7)	503.2 (6.0)	598.6 (6.3)	665.6 (6.4)	665.6 (6.5)	743.1 (6.7)	728.3 (6.7)	776.4 (6.8)
											
Indigenous status											
Indigenous	116.5 (10.2)*	171.3 (12.3)*	173.3 (12.2)*	496.8 (20.4)	536.2 (21.1)*	703.3 (24.0)*	741.5 (24.6)*	715.7 (24.1)*	800.9 (25.5)*	787.4 (25.3)*	806.6 (25.7)*
Non-Indigenous	220.2 (3.0)	287.3 (3.4)	261.3 (3.2)	455.0 (4.3)	438.8 (4.2)	562.1 (4.7)	609.5 (4.9)	621.2 (5.0)	708.9 (5.3)	693.9 (5.3)	727.7 (5.4)
											
Extraction 1+	109.2 (2.9)	145.9 (2.4)	129.0 (2.2)	263.8 (3.2)	267.9 (3.2)	408.9 (3.9)	446.0 (4.1)	445.7 (4.1)	519.5 (4.4)	499.8 (4.4)	540.0 (4.5)
											
Restoration 1+	139.5 (2.3)	177.1 (2.6)	162.1 (2.5)	300.1 (3.4)	299.7 (3.4)	358.1 (3.7)	382.6 (3.8)	402.9 (3.9)	452.5 (4.1)	448.0 (4.1)	462.6 (4.2)

*P < 0.05

Results from the Poisson regression are detailed in Table 2. We carried out 3 regressions; overall DGA rates, DGA rates in which receipt of one or more extractions were considered, and DGA rates in which receipt of one or more restorations were considered. After adjusting for other covariates, children who were aged 0–4 years, male, Indigenous or rural/remote-dwelling had higher overall DGA rates than their 5–9-year-old, female, non-Indigenous or metropolitan-dwelling counterparts respectively. After adjusting for other covariates, DGA rates in 2003–2004 were 3.4 times those in 1993–1994, with a linear trend being observed in the intervening years. For admissions in which one or more extractions were received, children aged 0–4 years had higher rates than 5–9-year-old children after adjusting for other covariates. The rate of males was 16% greater than for females, Indigenous rates were 46% greater than non-Indigenous rates and rural/remote-dwelling child rates were 18% greater than the rates of metropolitan-dwelling children. In 2003–2004, the DGA rate in which one or more extractions were received was 4.9 times that observed in 1993–1994. For admissions in which one or more restorations were received, children aged 0–4 years had higher rates after adjusting for other covariates. The rate of males was 8% greater than for females, Indigenous rates were 10% greater than non-Indigenous rates and rural/remote-dwelling rates were 8% greater than metropolitan-dwelling rates. In 2003–2004, the DGA rate in which one or more restorations were received was 3.3 times that observed in 1993–1994.

**Table 2:** Poisson regression models for overall DGA rates, DGA rates in which 1+ extractions were received and DGA rates in which 1+ restorations were received

	DGA RATES OVERALL	DGA RATES with 1+ EXTRACTION	DGA RATES with 1+ RESTORATION
	Rate ratio (95% CI)	Rate ratio (95% CI)	Rate ratio (95% CI)

Age group			
0–4 years	1.09 (1.08–1.10)	1.11 (1.10–1.13)	1.28 (1.26–1.29)
5–9 years (ref)	1.00	1.00	1.00
			
Sex			
Male	1.11 (1.10–1.12)	1.16 (1.15–1.18)	1.08 (1.07–1.10)
Female (ref)	1.00	1.00	1.00
			
Indigenous status			
Indigenous	1.09 (1.06–1.11)	1.46 (1.43–1.50)	1.10 (1.07–1.11)
Non-Indigenous (ref)	1.00	1.00	1.00
			
Location			
Metropolitan (ref)	1.00	1.00	1.00
Rural/Remote	1.10 (1.09–1.11)	1.18 (1.16–1.20)	1.08 (1.06–1.10)
			
Year			
1993–1994 (ref)	1.00	1.00	1.00
1994–1995	1.31 (1.26–1.35)	1.05 (0.99–1.10)	1.27 (1.21–1.33)
1995–1996	1.19 (1.15–1.24)	1.18 (1.12–1.24)	1.16 (1.1–1.22)
1996–1997	2.12 (2.05–2.19)	2.41 (2.31–2.52)	2.15 (2.07–2.24)
1997–1998	2.05 (1.99–2.12)	2.45 (2.34–2.56)	2.15 (2.07–2.24)
1998–1999	2.64 (2.56–2.72)	3.73 (3.58–3.89)	2.57 (2.48–2.67)
1999–2000	2.86 (2.77–2.94)	4.07 (3.91–4.24)	2.75 (2.65–2.86)
2000–2001	2.90 (2.82–2.99)	4.07 (3.90–4.24)	2.90 (2.79–3.01)
2001–2002	3.31 (3.21–3.41)	4.74 (4.55–4.94)	3.25 (3.14–3.38)
2002–2003	3.24 (3.14–3.34)	4.56 (4.38–4.75)	3.22 (3.10–3.35)
2003–2004	3.39 (3.29–3.50)	4.93 (4.73–5.13)	3.33 (3.21–3.45)

## Discussion

This study has shown that trends in DGA receipt among Australian children are increasing. Pre-school, male, Indigenous and rural/remote-dwelling children are at higher risk of receiving such care. There is a propensity towards extraction as opposed to more conservative procedures, with Indigenous DGA rates being 46% higher when procedures in which one or more extractions were received were considered.

Before interpreting our findings, it is important to consider the weaknesses of the study approach. Our investigation was essentially a secondary analysis of routinely-collected data. While such analyses may be useful in providing preliminary estimates of the magnitude of a suspected problem, investigators have limited control over what information is collected and the way in which it is stored. For example, our study lacks important data pertaining to waiting times, the rate of children not presenting for care, follow-up of care, repeat DGAs and clinical measures such as the prevalence and severity of dental disease experience. The study also relies on data being collected by hospital administrators who may not, for whatever reason, be as rigorous in the data coding and cleaning processes as researchers with specific aims and objectives in mind.

Taking into account these caveats, our findings raise a number of questions. The most important is perhaps why, given that the proportion of children with dental disease experience in Australia has decreased, or at least stabilized, in the last 10 years, have child DGA rates increased? Possible speculations include: improved enumeration of data by hospital administrative personnel, increased number of paedodontists (whose preferred modality of treatment may be a general anaesthetic), increased resources for DGA sessions, reluctance of dentists to treat children with complex dental presentations in dental chair settings, not enough dental personnel to treat high-risk children in the preventive stages of dental disease, consumer demand, an increase in the severity of dental disease among certain child groups or rising fears of litigation among those who may have previously performed DGA procedures in non-hospital settings.

That DGA rates were higher among pre-school children in our study was unsurprising given evidence in the literature that suggests that children in this age-group have more behaviour problems in the chair [9] and that parents of this group are more supportive of dental care being provided under hospital general anaesthetic settings as opposed to under local anaesthesia [10]. It is a public health concern, however, given evidence that children requiring DGA care at a young age are at higher risk of repeat DGA procedures in later life, with ongoing dental morbidity throughout the lifecourse [11]. The prevalence of dental fear is also common among children who received DGA care at a young age [12]. Children in Australia can enroll in the School Dental Service (SDS) at any age, but the Service typically treats children aged 5+ years. This means their younger counterparts may not receive the care they require until such an age when the dental disease cannot be treated by simple conservative procedures.

Evidence suggests that male children in Australia, on the whole, have higher levels of dental disease experience in the primary dentition than female children [13]. This may have been a reason why males had higher rates of hospital admissions for dental procedures relative to their female counterparts in our study. However, elsewhere in the literature there do not appear to be disparities in DGA receipt in relation to gender [4,14]. Further investigations to explore this disparity may prove valuable.

It was unsurprising that Indigenous child DGA rates were higher than their non-Indigenous counterparts, given the greater prevalence and severity of dental diseases among the Indigenous child population compared with non-Indigenous children [15]. The relative autonomy of Indigenous children in comparison with non-Indigenous children [16] and general reluctance of Indigenous children to attend for dental care may mean such children do not receive the treatment they require until their oral health presentation progresses to a more severe stage [17]. Indigenous children in Australia are disadvantaged on many levels; historical, political, societal, economical and health [18]. It could be that Indigenous disparities in receipt of DGA care (along with many other health outcomes) will persist until such fundamental upstream factors are addressed.

Rural/remote-dwelling children may have had higher rates of DGA care than their metropolitan-dwelling counterparts due to disparities in access to dental services. Children in some rural communities are visited by the SDS less than once every two years, whereas their urban counterparts may receive dental care more frequently [19]. Medical personnel employed in remote community health clinics frequently have limited oral health training and are thus unable to provide care other than referral for dental appointments when a dental health professional is next in the community. Vast geographic distances between rural townships in Australia and limited dental personnel in remote communities mean rural-dwelling caregivers may put off dental care for their children until it can no longer be ignored; meaning teeth with carious lesions that may have been treated conservatively in the early stages require more radical treatment once the infection has spread to a tooth's innervation system (when a child most typically feels pain). Rural-dwelling caregivers may have poor oral health themselves, with caregiver's dental disease experience being a strong predictor of child caries experience [20]. Rural-dwelling children are, on the whole, more socio-economically deprived than metropolitan-dwelling children [21], with the association between low socio-economic status and dental disease experience being well established [22].

It is of concern that rates of extractions increased so markedly in the observation period (a 4.9-fold increase from 1993–1994 until 2003–2004 after adjusting for covariates), given the impact extractions may have on the remaining dentition and psychological health. Untimely extraction of deciduous teeth may lead to earlier-than-normal eruption of permanent teeth, with displacement likely, while extraction of permanent teeth may lead to over-crowding and drifting of remaining teeth if no prosthesis is placed [23]. Psychological and speech disorders may also occur, especially with removal of anterior teeth [24].

There are many reasons that a child may receive an extraction as opposed to more conservative restorative treatment when receiving DGA care, including extensive dental pathology, time restrictions or reluctance for a repeat procedure should restorative care prove unsuccessful. Although it was not possible to ascertain if children who received extractions had similar oral health presentations as those who received more conservative care in our study, the literature indicates that – even when dental disease experience is equal – children from socially-deprived backgrounds receive more extractions and less restorations or preventive care under a general anaesthetic than their more affluent counterparts [6,25,26]. Some children may have also received higher rates of extractions due to provider bias and expectations. Treatment decisions made by dentists who provide DGA care are complex, but it is apparent that clinical considerations are not the only factors that influence the treatment provided. Although unexamined in the Australian context, investigations in the United Kingdom have revealed that dental practitioners selectively choose to restore some teeth whilst leaving other carious teeth untreated [26-28] and Tickle et al. found that dentists were differentially prescribing prophylactic extractions (extractions other than those for pain and sepsis), for poorer children [29].

Expectations of carers also play a role in the type of DGA treatment a child receives, with requests for teeth to be retained or removed influenced by familial access to dental services, a child's compliance with oral hygiene, a child's behaviour in the dental chair, oral health experience of other family members, priority of oral health to family members and familial dental health awareness [30]. Hood and colleagues reported that carers of children from more affluent backgrounds were more likely to demand conservative care than tooth removal, whereas parents from deprived backgrounds were more likely to accept extractions [6]. Given that restorative care is almost always preferable to extractions, it may be important for hospital personnel to explore greater opportunities to carry out conservative procedures for any child undergoing DGA, an assertion supported by Hosey and others [4].

## Conclusion

Our study has shown that child DGA rates in Australia are increasing and that disparities exist in relation to age, sex, Indigenous status, location and type of care received. Reasons for our findings are likely to be complex but may include barriers in access to care, limited resources, high treatment needs, care-giver preference, treatment bias by service providers, time limitations and behavioural factors. More research is required to better understand the public health implications of increasing trends in child DGA care and how disparities in the receipt of such care might be reduced. The findings have public health and dental service provision relevance both in Australia and other developed and developing nations.

## Competing interests

The author(s) declare that they have no competing interests.

## Authors' contributions

LMJ obtained and analysed the data from the Australian Institute of Health and Welfare Hospital Morbidity Database, and drafted the manuscript. KRT participated in the design of the study and completion of the manuscript. All authors read and approved the final manuscript. There were no sources of funding for this study.

## Pre-publication history

The pre-publication history for this paper can be accessed here:


